# Real‐World Implementation of Contingency Management and Benefits of a Controlled Environment “Head Start”

**DOI:** 10.1002/brb3.70710

**Published:** 2025-09-08

**Authors:** Himmat Singh Dhillon, Stephanie Rochon, Basant Kaur Dhillon, Ahmed N. Hassan, Timothy H. Guimond, Tanya S. Hauck

**Affiliations:** ^1^ Family Medicine Residency Creighton University Phoenix Arizona USA; ^2^ Norfolk General Hospital Simcoe Ontario Canada; ^3^ Health Sciences McMaster University Hamilton Ontario Canada; ^4^ Department of Psychiatry University of Toronto Toronto Ontario Canada; ^5^ Centre For Addiction and Mental Health Toronto Ontario Canada; ^6^ Department of Psychiatry King Abdul Aziz University Jeddah Saudi Arabia; ^7^ Department of Pharmacology and Toxicology, Faculty of Medicine University of Toronto Toronto Ontario Canada; ^8^ Institute of Medical Sciences University of Toronto Toronto Ontario Canada; ^9^ ICES Toronto Ontario Canada; ^10^ Institute of Health Policy, Management and Evaluation University of Toronto Toronto Ontario Canada

**Keywords:** contingency management, quality improvement, rapid access addiction aedicine (RAAM) clinic, stimulant use disorder

## Abstract

**Objectives:**

To use quality improvement (QI) principles to implement contingency management (CM) in an outpatient clinic.

**Methods:**

Prize‐based CM was implemented for stimulant use disorder using standard protocols with QI processes used to improve efficiency and effectiveness.

**Results:**

CM was successfully implemented in an outpatient addictions clinic using clinical funds. Participants who were discharged to the outpatient clinic from a controlled environment (such as hospital, withdrawal management, residential treatment program) had 8.3 (*SD* = 3.1) weeks of consecutive abstinence (CA) and 10.7 (*SD* = 1.0) weeks total abstinence.

**Conclusions:**

Pragmatic clinical implementation of CM yielded results that are comparable to controlled trials in similar populations. QI processes identified that a controlled environment resulted in significant ongoing abstinence.CM was implemented in a small community clinic with no additional funding, and QI principles were used to improve efficiency and effectiveness. The initial implementation of 17 participants yielded a mean of 2.2 (*SD* = 3.2) weeks of CA. Recent discharge from a controlled environment resulted in 8.3 (3.1) weeks CA.

## Graphical abstract

Contingency Management (CM) was implemented in a small community clinic with no additional funding, and Quality Improvement (QI) principles were used to improve efficiency and effectiveness. The initial implementation of 17 participants yielded a mean of 2.2 (SD 3.2) weeks of consecutive abstinence (CA). Recent discharge from a controlled environment resulted in 8.3 (3.1) weeks CA. Electronic Medical Record (EMR).

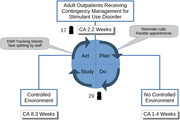

## 1 Background

Stimulant use disorder (SUD) is an increasing concern in North America (Groot et al. 2016) and has no on‐label pharmacological treatments. However, a Cochrane review identified CM as the most promising psychosocial intervention (Minozzi et al. 2016) and CM is the “current standard of care” according to recent treatment guidelines. (Clinical Guideline Committee (CGC) Members et al. 2024) CM uses rewards based on measurable behaviors, such as urine drug testing results, to reinforce recovery (Petry 2013). CM has extensive evidence for stimulant use disorder, (Silverman et al. 1996) and a large effect size among psychosocial treatments (Petry et al. 2017; Bentzley et al. 2021). There are standard protocols for CM, and it is considered safe although there are concerns about increasing gambling behaviours (Petry 2013). However, despite the large number of studies supporting CM, it is not widely used, and available data is largely from well‐funded controlled trials and not pragmatic community implementation (Miguel et al. 2016). There are many barriers to CM implementation, including cost, ethical objections and lack of experience with CM.

Compared to prescribing a medication or providing psychotherapy such as relapse prevention or cognitive behavioural therapy, CM implementation involves training, staffing, logistics, and funding, making it particularly suited to QI processes. QI is a systematic approach to problem solving in health care, in which a particular metric is targeted (such as safety, effectiveness, experience of care) by understanding the health care environment and then designing, testing, and implementing process changes while continuously measuring outcomes (Jones et al. 2019). This study seeks to assess the feasibility of implementing CM in a Canadian addiction clinic using clinical resources, and to determine if pragmatically implemented CM has comparable outcomes to standard CM from clinical trials. Our secondary goal is to improve effectiveness and efficiency of CM implementation through QI processes.

## 2 Methods

Given the evidence for CM in SUD and the lack of on‐label pharmacological treatments, the Brant Haldimand Norfolk Rapid Access Addictions Medicine clinic (BHN RAAM) in Ontario, Canada decided to implement CM for SUD in January 2020. SUD is a common presenting concern at the clinic, with many patients concurrently receiving opioid agonist treatment or presenting for treatment of alcohol use disorder. RAAM clinics are outpatient addictions clinics developed in Ontario, Canada, providing urgent, non‐appointment based care for substance use disorders, (Srivastava et al. 2021) and are a particularly relevant setting for the implementation of accessible, low‐barrier treatments like CM.

In 2022 we embarked on a QI process to examine the effectiveness of this implementation compared to the literature and to improve the delivery of the service. The project was approved by the Centre for Addiction and Mental Health Quality Projects Ethics Review (2022_017) and Brant Community Healthcare System Research Ethics Committee. No research funding was received for this project, and CM was provided as part of evidence‐based addictions care.

A standard prize‐based “fishbowl” CM protocol was implemented, (Petry 2013), involving twice‐weekly immunoassay urine testing for stimulant drugs (benzoylecgonine, amphetamines, methamphetamine) and immediate provision of draws for prizes for negative samples. CM can be used to target any substance of concern or other measurable targets (such as attendance), but we were targeting only stimulant drugs in our program. This is because first‐line, on‐label treatment for alcohol and opioid use disorder are readily available in our community, but there are very few available treatment programs for stimulant drug use disorders. CM is also well‐tested and protocols are well‐developed specifically for stimulant drugs.

The protocol was the same for both rounds of the protocol, and followed the guidance in the standard CM manual (Petry 2013). The approximate cost of prizes and tests per participant was $250–300 (Canadian). This is an approximation as prize costs fluctuated and it was a pragmatic implementation involving donations and shifting bulk purchases of prizes, as there was no formal funding to establish the program. In both rounds, participants earned up to eight draws per day for eight or more consecutive negative samples (Supplementary Figures 2 and 3), with twice‐weekly testing for twelve weeks. Prizes available included small, medium, large and jumbo prizes in the standard ratio described in the manual. Examples of prizes included candy or small toiletries for small prizes, household items such as coffee tumblers or frying pans for medium prizes, small appliances such as coffee makers for large prizes, and one or two jumbo prizes like electronics or tablets. Prizes fluctuated based on patient feedback, availability, and donations. Supplementary Figure 4 shows how a prize is recorded.

Inclusion criteria were adults 16 + with established SUD and a goal of stimulant abstinence, interest in the program, on a first‐come first‐served basis; and exclusion criteria were prescription stimulant use during the treatment period or a history of gambling disorder. Patients signed a treatment agreement and were categorized as round 1 (R1) from the 2020 implementation and round 2 (R2) from the 2022 implementation and QI process.

Data were collected in a secure EMR system (Avarros) and anonymized. Demographic data collected included age (five‐year increments), self‐identified gender, and employment status. At each of the 24 visits, absences, drop‐outs, and UDS results were recorded, as well as selected prizes. In the second round, recent admission to a controlled environment (prison, hospital, psychiatric hospital, withdrawal management, or residential treatment) was measured if the patient was enrolled in CM within three days of discharge and initial UDS was negative. Data was analyzed in R (version 4.2.1) using the tidy verse package. Continuous data were assessed for normality by q‐q plots and Kolmogorov–Smirnov tests, summarized using means and standard deviations, and compared with the Wilcoxon Rank Sum Test. Categorical data were summarized with frequencies and percentages, and compared using Fisher's exact test.

Significant QI interventions involved outcomes of (1) identifying and addressing reasons for drops outs; (2) embedding workflows for CM into the available human resources capacity; and (3) assessing the relevance of starting CM after leaving a controlled environment to maintain abstinence from stimulant use.

## 3 Results

Results are summarized in Table 1. There were 17 participants (Round1 = R1) in 2020 and 29 participants (Round2 = R2) in 2022. The age distribution of participants is shown in Supplementary Figure 1. Three patients dropped out in R1 (17.6%) and four dropped out in R2 (13.8%), with the majority of drop outs (6/7) leaving within the first quarter of the treatment (by session 6) and only one person dropping out later than that. While consecutive weeks of abstinence and total weeks of abstinence were collected for each individual as an integer valued response, the data overall appears normally distributed. However, when divided into subsamples qq‐plots were less consistent with a normal distribution. Consecutive weeks of abstinence was similar, with 2.2 (*SD* = 3.2) in R1 and 3.1 (*SD* = 3.5) in R2, and total weeks of abstinence were 2.6 (*SD* = 3.5) in R1 and 4.6 (*SD* = 4.0) in R2. Patients who left a controlled environment immediately prior to the intervention (*n* = 7) had significantly higher CA (8.3 (SD = 3.1) versus 1.4 (*SD* = 1.2) weeks) and significantly higher total abstinence (10.7 (*SD* = 1.0) versus 2.6 (*SD* = 2.1) weeks).

**TABLE 1:** Consecutive and total weeks of abstinence among participants. Results are given as means and standard deviations. CE: Controlled environment; NCE: Non controlled environment *Due to the small sample size and small community, identifying details such as exact age werenot collected to identify data, only age range was recorded. As a result, it is not possible to calculate a median age. For the same reason, we were unable to record participation in opioid agonist therapy on an individual level.

–	Round 1 (2020)	Round 2 (2022)		Difference
Participants	17	29		—
Self‐reported gender (man %)	76% (*n* = 13)	79% (*n* = 26)		*p* = 1.0
Age (mode)	35–40 years	30–34 years		*
Dropouts	3 (17.6%)	4 (13.8%)		*p* = 1.0
Consecutive abstinence (weeks)	2.2 (3.2)	3.1 (3.5)		*p* = 0.17
Total abstinence (weeks)	2.6 (3.5)	4.6 (4.0)		*p* = 0.067
Consecutive abstinence (weeks) by controlled environment	—	CE (*n* = 7)	NCE (*n* = 22)	*p* = 0.00008
		8.3 (3.1)	1.4 (1.2)	
Total abstinence (weeks) by controlled environment	—	CE (*n* = 7)	NCE (*n* = 22)	*p* = 8.5 × 10^−5^
		10.7 (1.0)	2.6 (2.1)	

Please refer to Supplementary Information and Supplementary Table 1 for a breakdown of the QI processes, measures, analyses and results. QI processes identified strategies for reducing dropouts and maximizing scarce human resources based on our experience in the first round. In R1, it was recognized that dropouts were happening early in treatment. In R2, reminder calls were implemented particularly in the first few weeks to reduce drop outs. In addition, set appointment times had been used in R1, and this was challenging for participants and led to a drop in model in R2.

In terms of human resources, there are a number of options in terms of allocating the new workload associated with implementation of CM. In this clinic, the most efficient system was to separate the CM care such that one staff member does the urine testing and another helps the client select prizes and manage inventory.

## 4 Discussion

Total and consecutive weeks of abstinence in this pragmatic CM implementation are comparable to 2.8 weeks of abstinence found in a population also treated with methadone (similar to our population), although not as good as the 4.4 weeks of CA seen in randomized trial data for stimulant use disorder (Petry et al. 2017; Benishek et al. 2014). Implementation without the clinical trial infrastructure is expected to result in reduced effectiveness, and this study shows that it is feasible to implement CM in an outpatient setting, using existing clinical resources, with comparable results.

Important recent work has demonstrated the widespread pragmatic implementation of CM, such as in Veterans Affairs (DePhilippis et al. 2023). However, it is important to note that in many worldwide community clinics such as ours, there is no allocated funding to implement CM, even though it is first‐line evidence based treatment. This study demonstrates what local implementation can look like, when there is no regional or national program that one can join, or additional funding available. Given the present healthcare funding and human resources challenges, QI optimization of resources is crucial in delivering the most services to the most patients. We would expect that each clinic has different resources and staffing, and the use of QI processes may allow individual clinics to implement this intervention. For example, in our clinic, the use of two staff members reduced the time needed to provide the service, whereas in other settings one staff member may be able to provide CM on their own.

This QI initiative illustrated that the timing of CM relative to a controlled environment is important, and we were able to find only very limited consideration of QI timing (before, during or after residential treatment) in the literature (Godley et al. 2014). Important work has demonstrated that abstinence status at the time of initiating CM is critical and predicts outcomes and the amount of reinforcement needed to promote ongoing abstinence (Alterman et al. 1997; Petry et al. 2012; Regnier et al. 2023). However, it is not clear how discharge from a controlled environment and the use of CM directly as “aftercare” may be particularly effective. There were notable differences in abstinence when patients had an established SUD but enter the program after a controlled environment “head start”. This merits further research as there are often waiting periods between treatment services, such as withdrawal management, residential treatment, and aftercare services. Many clients attend withdrawal management and then have to go back to their housing environment or shelter and wait over a month for residential treatment; and CM may help bridge this gap. Relapse after residential treatment for cocaine use is also very common, and CM may be a cost‐effective aftercare or transitional bridging program in addition to routine care.

There are several limitations to this study. It is observational, and there was no assignment to a controlled environment. The sample size is small, and the first round of the study was mid‐way when the COVID‐19 pandemic was declared, although the BHN RAAM remained open for in‐person care throughout the pandemic.

## 5 Conclusions

CM is a safe, underutilized, and can be pragmatically provided in the community. QI optimization of CM implementation can overcome barriers such as staffing, which have prevented more widespread use of this effective treatment. QI processes identified treatment timing as a key issue, and CM was more effective when offered to patients after planned or unplanned periods in a controlled environment. CM may be more effective in sustaining rather than initiating abstinence, and future work is essential to assess the utility of CM as aftercare.

## Author Contributions

**Himmat Singh Dhillon**: writing–review and editing, writing–original draft, conceptualization, project administration, investigation. **Stephanie Rochon**: conceptualization, investigation, writing–review and editing, project administration, resources, supervision. **Basant Kaur Dhillon**: project administration, investigation. **Ahmed N. Hassan**: conceptualization, writing–review and editing. **Timothy H. Guimond**: conceptualization, formal analysis, writing–review and editing. **Tanya S. Hauck**: conceptualization, investigation, writing–original draft, writing–review and editing, visualization, validation, methodology, formal analysis, project administration, supervision, data curation.

## Conflicts of Interest

The authors declare no conflicts of interest.

## Peer Review

The peer review history for this article is available at https://publons.com/publon/10.1002/brb3.70710

## Supporting information




**Supporting Material**: brb370710‐sup‐0001‐SuppMat.docx

## Data Availability

The data that support the findings of this study are available on request from the corresponding author. The data are not publicly available due to privacy or ethical restrictions.
