# Supplementary material for: Real‐World Implementation of Contingency Management and Benefits of a Controlled Environment “Head Start”
Source: Brain Behav. 2025 Sep 8;15(9):e70710. doi: 10.1002/brb3.70710 (PMC12417560; doi:10.1002/brb3.70710)
Supplement: Supplementary file 1 — Supporting Material: brb370710‐sup‐0001‐SuppMat.docx [file BRB3-15-e70710-s001.docx]

# Supplementary Data

Real-world implementation of contingency management and benefits of a controlled environment “head start”

Himmat Singh Dhillon BSc, Stephanie Rochon RPHT, Basant Kaur Dhillon, Ahmed Hassan, MD MPH, Tim Guimond MD, PhD, Tanya Hauck MD, PhD*

## Additional Background on RAAM Clinics:

RAAM clinics are unique low-barrier drop-in models for substance use and stimulant use disorder (SUD) is a common presenting concern, with few available treatment options. Patients often delay seeking treatment for substance use due to long wait times; however, the drop in model of RAAM appointments allow patients to be seen on the same day. RAAM serves as an anchor, as patients with SUD often lack a primary care provider (PCP) and their first access to care is through RAAM.

## Costs of the Program:

We estimate that a 12-week course of CM in the clinic costs about $250-300 (Canadian dollars) in prizes and equipment, although this depends on the types of prizes selected and whether any prizes are obtained by donations from the community.

## Considerations of Controlled Environment Discharge:

Many patients (including RAAM patients) seeking treatment for stimulant use are interested in both CM and residential treatment–a 30-day inpatient treatment. Current literature does not indicate whether CM should be administered before or after residential treatment, which serves as a barrier to optimal patient care. This has clinical implications as it questions whether CM is better at initiating or maintaining abstinence. In other words, do patients who come from residential treatment (and hence would be stimulant negative starting CM) achieve more success on the program (more weeks of abstinence) compared to an individual starting CM without initial abstinence. CM has traditionally been thought of as a way to initiate abstinence; however, it may be actually better at maintaining it. Through an observational approach, this is something we investigate in our study and points to the need for further randomized controlled trials to study this.

The Brant Haldimand Norfolk RAAM patient population frequently presents to the clinic from prison, hospital, psychiatric hospital, withdrawal management (“detox”) or residential treatment. Patients coming from these environments would potentially (although not always) be stimulant negative as access to stimulants is reduced in these environments. In using QI principles in this study, the question of how to time CM with respect to a controlled environment was raised by patients and providers.

In the study, patients were designated as a controlled environment (CE) if they presented to the clinic within 3 days of discharge and confirmed to be negative prior to CM start via a urine immunoassay analysis. The objective of this was to see if patients who wanted to maintain abstinence coming from these CEs would achieve better results than a patient who wanted to start CM to initiate abstinence (stimulant positive starting the program). If individuals achieved better success coming from a CE this would suggest CM is more effective at maintaining abstinence rather than initiating it and would inform provider decision making by recommending residential treatment prior to CM for best results.

## Additional Details on the Quality Improvement Process:

## Dropouts

In R1, we noticed that patient dropouts occurred within the first few weeks and occurred in those who missed the first 1-2 sessions of CM. We concluded that early CM engagement is likely necessary to promote program attendance. So, in R2, a prominent strategy that reduced the number of dropouts included reminder phone calls to CM patients especially during the first few weeks. Also, in R1, there were set appointment times on specific dates for CM. Following a strict schedule is difficult for patients with stimulant use and for patients who work. After studying this effect, in R2, patients could attend their CM program any time during clinic hours on Tuesdays and Fridays, making patient adherence higher. Both improvements are generalizable to any clinic engaging in CM.

## Human Resources Optimization

Equal division of CM responsibility was critical to avoid staff burnout and permit flexibility in the event of staff absences. Two staff members splitting the work led to best results as one completed point of care urine tests and entered results (laboratory technician) and one assisted the client with picking prizes and updating inventory (case manager). This is a feasible and natural division of labor in this setting as the laboratory technician would already be engaging in urine immunoassay analysis from other clients (such as clients receiving methadone) and case managers follow up the patients after urine results are in and provide psychosocial support.

Electronic health records and documentation requirements are a frequently cited contributor to staff burnout and take considerable resources. It was essential to use electronic medical records (EMR) in a flexible and efficient way to promote efficiency. Our clinic used Avarros EMR which populated CM patient appointments in a distinct color, allowing staff to readily identify who is in the CM program, and this facilitated prompt follow up for missed appointments and rapid recording of missed appointments. In addition, Avarros allowed us to use and fill electronic forms uploaded into the EMR to optimize CM session tracking (Supplementary Figure 4).

## Statistical Considerations and Limitations

The small sample size may also make it difficult to confirm normality or non-normality of the distribution of data. However, the t-test tends to be a robust test to violations of the normality assumption so long as there are not serious outliers that might lead to a violation of the finite variance assumption, a condition that is met due to the upper limit for this data in the number of weeks abstinent. Replication of these findings with a larger dataset would provide further reassurance.

**References for R:**

R: A language and environment for statistical computing. R Foundation for Statistical Computing. https://www.R-project.org/.

Wickham H AM, Bryan J, Chang W, McGowan LD, François R, Grolemund G, Hayes A, Henry L, Hester J, Kuhn M, Pedersen TL, Miller E, Bache SM, Müller K, Ooms J, Robinson D, Seidel DP, Spinu V, Takahashi K, Vaughan D, Wilke C, Woo K, Yutani H. Welcome to the tidyverse. Journal of Open Source Software, . 2019;4(43).

**Supplementary Figure 1.** **Age Distribution of CM Participants in R1 vs R2**

**
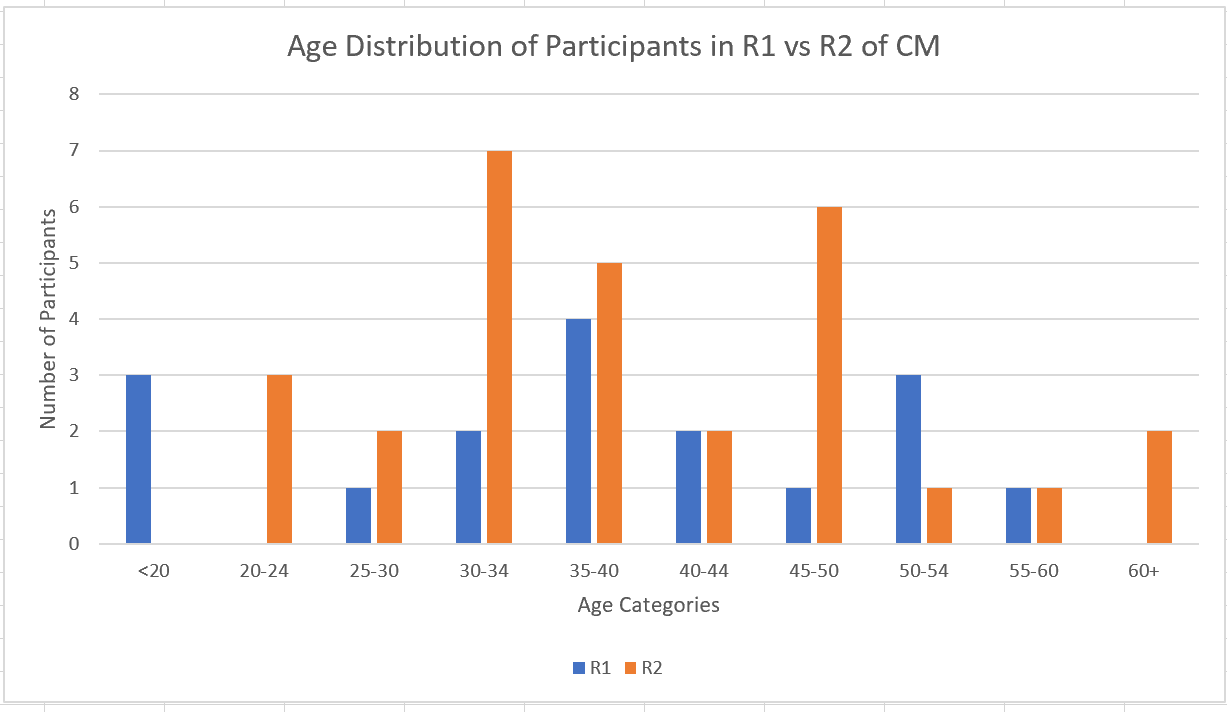
**

**Supplementary Figure 2.** Protocol schematic of standard CM prize-based protocol.

**
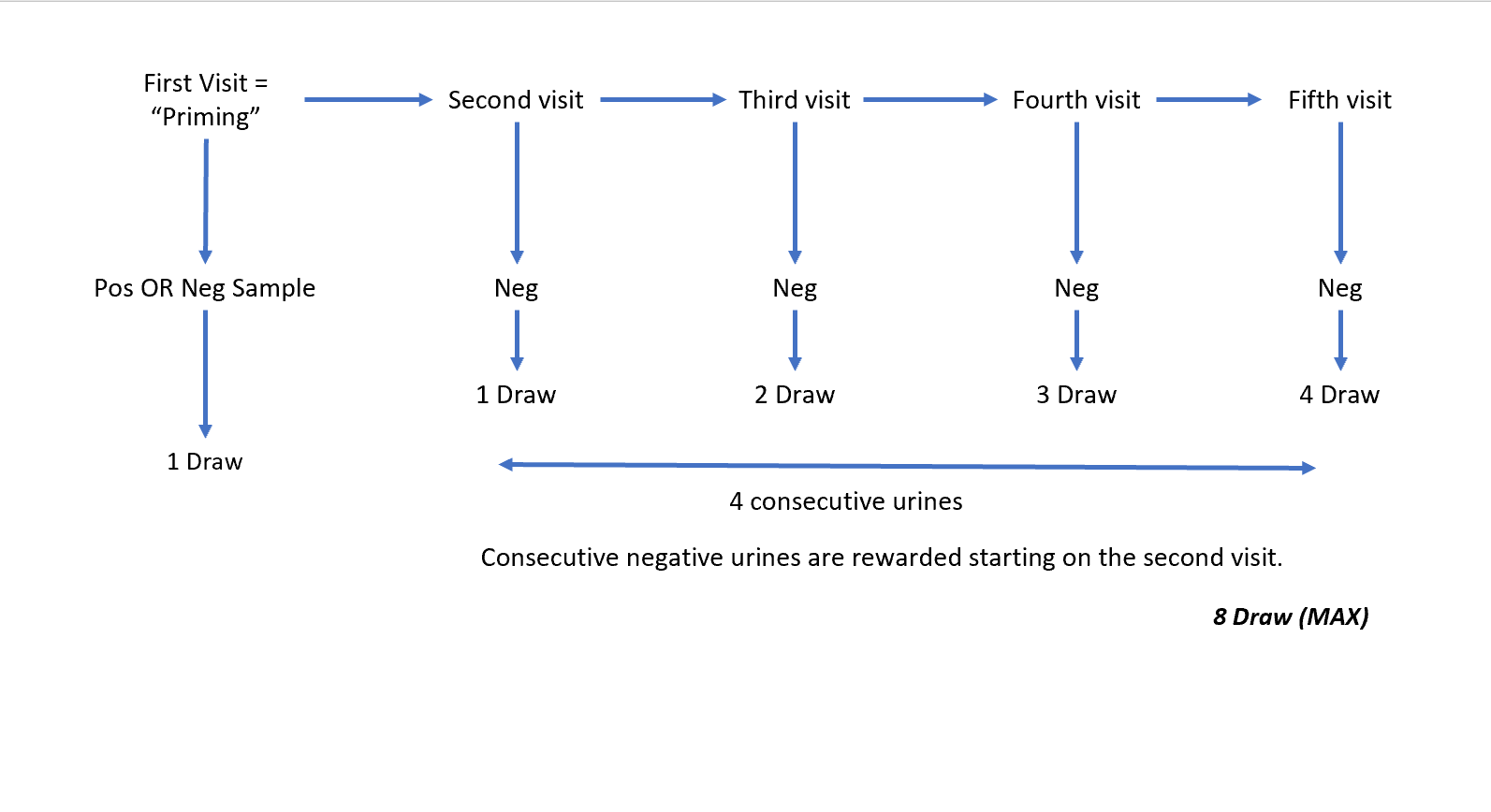
**

**Supplementary Figure 3.** Protocol schematic example for a positive urine sample.

**After priming, participants earned one draw for each consecutive negative sample, to a maximum of eight draws a session. For example, on the day of their eight consecutive negative sample they would draw eight times out of the prize bowl, and on the day of their ninth consecutivd negative sample it would still be only eight draws. Any non-negative samples would reset back to zero draws.**

**Supplementary Figure 4.** Electronic form for recording results of a single session (simulated example)


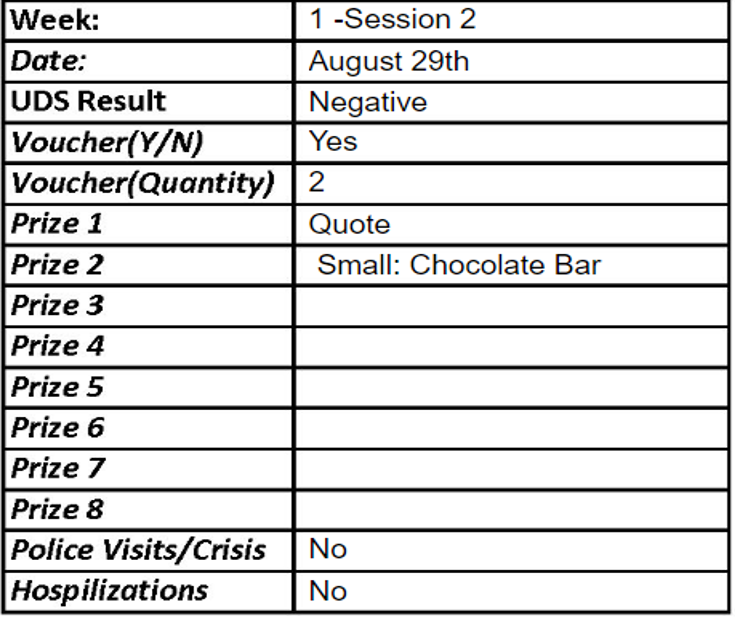


| **Problem** | **Intervention** | **Measures and Analysis** | **Results** |
| --- | --- | --- | --- |
| Patients dropping out of care (missed the first 1-2 sessions of CM) | (1) Reminder calls to patients  (2) Using EMR to create appointments, although patients could come at any time on Tuesdays and Fridays | Dropouts were measured through EMR.  Staff surveyed about how patients were responding to appointments and reminders. | No change in dropouts between R1 and R2, although process implemented throughout R2.  Improved ability to track all 24 visits in EMR.  Reinforcement Reminder Forms from the protocol were abandoned because patients did not like them this created a problem with litter in the clinic. |
| Human resource needs exceed available staff | (1) Use of a new tracking sheet in EMR software  (2) Task splitting between various staff, such as one staff doing immunoassay urine tests and another staff doing prize allocation. | Time per patient per week was calculated and optimized.  Staff were involved in iterative assessment to incorporate feedback about feasibility of processes. | Process was optimized to require 10 minutes per patient, twice per week.  Ability to predict staffing needs based on data collected allowed better planning and human resource measurement. |
| Optimal timing of CM intervention (relative to residential care or withdrawal management) | (1) Tracking of whether or not patients came to CM within three days of a controlled environment and a negative urine screen for stimulants | Total and consecutive weeks of analysis were measured and analyzed, compared to patients not coming from a controlled environment. | As documented in table 1, significantly higher total and consecutive weeks of abstinence observed for patients leaving a controlled environment. |

Supplementary Table 1: Quality improvement results and processes.
